# Nipping Diseases in the Bud? Ethical and Social Considerations of the Concept of ‘Disease Interception’

**DOI:** 10.1093/phe/phaa036

**Published:** 2021-03-15

**Authors:** Jonas Narchi, Eva C Winkler

**Affiliations:** 1 National Center for Tumor Diseases (NCT) Heidelberg; 2 National Center for Tumor Diseases (NCT), Section of Translational Medical Ethics, University Hospital, Heidelberg

## Abstract

‘Disease interception’ describes the treatment of a disease in its clinically inapparent phase and is increasingly used in medical literature. However, no precise definition, much less an ethical evaluation, has been developed yet. This article starts with a definition of ‘disease interception’ by distinguishing it from other preventions. It then analyses the ethical and social implications of the concept in light of the four principles of medical ethics by Beauchamp and Childress. The term ‘disease interception’ refers to a form of secondary prevention applied in a short interception window intended to prevent a preclinical disease from developing further. We propose the definition ‘early and targeted secondary prevention by treatment’. The ethical evaluation of the concept shows that while it promises to be beneficial, it raises a number of ethical and social challenges regarding patient autonomy and justice. In order to ensure decision-making that respects patient autonomy, commercially motivated metaphors such as ‘disease interception’ should make way for precise definitions. Future research should not only focus on how to detect clinically inapparent diseases but also on the ethical question, when this is justifiable and what consequences it has for the individual and society as a whole.

## Introduction

Detecting, treating and curing manifest diseases before the patient develops any symptoms at all almost sounds too good to be true, but it is precisely what so-called ‘disease interception’ promises. What is the vision behind this new medical concept? Is it actually that new? What ethical aspects of the concept can be identified in a first *ex ante* analysis? What implications does it convey for our understanding of disease and for ‘healthy ills’?

In everyday language, the term ‘interception’ has primarily been used in ball sports where it indicates that a player of the opposing team interrupts a pass and catches the ball which would have otherwise reached the targeted player. It is also used in the context of telecommunications, e.g. when telephone calls are wiretapped. In medical research, the term ‘disease interception’ is relatively young and just beginning to gain more momentum—especially in cancer and neurodegenerative research. A Medline search results in only eight publications that use the term in the title—three of them with authors from one pharmaceutical company (Blackburn, 2011; Hait and Lebowitz, 2016; Beane *et al.*, 2017; Khan *et al.*, 2017; Walsh *et al.*, 2017; Dubinett and Spira, 2018; Albini *et al.*, 2019; Kampylafka *et al.*, 2019). Recently, the company has initiated the publication of the first volume of essays on the subject (Jessen and Bug, 2019) and even proclaimed a ‘paradigm shift’ in medicine (Klosterkötter, 2019). In these works and especially in the pharmaceutical public relations documents, the intriguing term ‘disease interception’ is used for reporting new treatment strategies for chronic diseases such as cancer and degenerative diseases. In these, there appears to be a therapeutic window at an early stage where treatment can prevent the irreparable consequences of the disease: the genesis of cancer (Blackburn, 2011; Beane *et al.*, 2017; Dubinett and Spira, 2018; Albini *et al.*, 2019), the destruction of nerve cells (Alzheimer’s dementia) (Walsh *et al.*, 2017; Albini *et al.*, 2019), the destruction of joints (rheumatoid arthritis) (Raza and Filer, 2015; Cossu *et al.*, 2017; Cole *et al.*, 2018) or the loss of islet beta-cell function (diabetes) (Insel *et al.*, 2017; Merino *et al.*, 2018). For these cases, the picture of ‘interception’ seems fitting, as in the case of the player, who intercepts the ball and thus interrupts the determined and predictable trajectory of its flight. This picture is novel, exciting and promising—but so far it remains nothing but a picture.

In order to encourage an ethical discourse on the implications of ‘disease interception’, this paper will seek to first clarify the precise meaning of the concept. It thus starts with a definition of ‘disease interception’ by distinguishing it from other ways of disease prevention. It then continues by discussing some of its foreseeable ethical implications in light of the four principles of biomedical ethics (Beauchamp and Childress, 2013). Lastly, we point to social implications for our general understanding of disease and the ‘healthy ills’.

## Definition of ‘Disease Interception’ and Distinction from Other Concepts of Prevention

From a first terminological analysis of the limited literature, four criteria of ‘disease interception’ can be distilled:

1. the disease has already begun but is still clinically inapparent,
2. the disease will (almost) certainly develop its full clinical picture without intervention,
3. there is only a short window of opportunity to intercept (referred to as ‘interception window’) and
4. the intervention is highly individualized and not as broadly applied to the population as measures of primary and secondary prevention (such as vaccines or cancer screenings).

The last criterion raises the important question how, if at all, ‘disease interception’ differs from prevention. Is ‘disease interception’ really a new concept or just an attractive way of presenting an old one? To answer that question, it needs to be distinguished from other preventive measures.

On the basis of Caplan’s classic account of prevention (Caplan, 1964), the U.S. Preventive Services Task Force differentiates between primary, secondary and tertiary prevention and coined the following definition: ‘In a clinical setting, primary preventive measures are those provided to individuals to prevent the onset of a targeted condition […], whereas secondary preventive measures identify and treat asymptomatic persons who have already developed risk factors or preclinical disease but in whom the condition has not become clinically apparent. […] Preventive measures that are part of the treatment and management of persons with clinical illnesses […] are usually considered tertiary prevention […]’. (U.S. Department of Health and Human Services, 1996: xli) Applying the definition above it becomes clear that ‘disease interception’ is a preventive measure treating asymptomatic persons who have already developed preclinical disease but in whom the condition has not become clinically apparent. By definition then, ‘disease interception’ is therefore *secondary prevention* or more precisely *a specific form of secondary prevention*. In philosophical terms it could be said, that ‘disease interception’ belongs to the *genus* of secondary prevention. Since every definition is classically composed of the genus and the *differentia specifica*, we can now use the previously determined specific attributes (2) to (4) and combine them with this genus to compose the following definition of the concept:

The term ‘disease interception’ refers to a form of secondary prevention (e.g. by medication) applied in a short interception window and intended to prevent a preclinical disease from developing its full clinical picture. To use a more neutral wording and translate the commercially motivated metaphor of ‘interception’ into scientific terms, we propose to use ‘early and targeted secondary prevention by treatment’.^1^ As can easily be seen, this term lacks the brevity, vividness and novelty effect of the metaphor ‘disease interception’. The contrast is, of course, intended: It can serve to demystify the concept and ideally to nip a commercially motivated hype in the bud. This critical perspective is especially important since recent research on early detection shows that the growing scientific literature on this closely related field focuses disproportionally on its potential benefits while sometimes ignoring potential harms (Hofmann and Skolbekken, 2017). Our proposed term is rather dry and informative and is intentionally devoid of the appeal and suggestiveness of ‘disease interception’. At the same time, the term early and targeted secondary prevention by treatment is apt to stress the new conceptual and ethical aspects of secondary prevention that have to be considered and cannot be sufficiently described in the existing taxonomy of prevention: Whereas the initial definition of secondary prevention included measures that both ‘identify and treat’, much of the literature has since focused on the implications of the former, not the latter: Screenings and other measures of early disease *identification* have been widely discussed in the context of public health and public health ethics (Weed, 2004; Dawson and Verweij, 2007; Marckmann and In der Schmitten, 2014). There is a similar discussion focusing more on the early treatment of asymptomatic patients but the identification aspect seems to be the primary interest—so much so, that secondary prevention is often reduced to and seen as synonymous with screening measures. Early and targeted secondary prevention by treatment as a specific form of secondary prevention focusing on early and targeted prevention not by identification but *by treatment* deserves a closer conceptual and ethical look.

Although the definition of early and targeted secondary prevention by treatment seems rather clear, it can sometimes be difficult to say, if and when all the criteria introduced above are met. There are at least two challenges to the definition concerning its applicability. The first touches upon the certainty of the event: Are there diseases where the latent stages that will certainly or almost certainly develop the full clinical picture? The second challenge is closely connected to this epistemological problem: If that were the case, how could a feasible testing strategy be developed to identify the early stage of a disease within the short pre-symptomatic window for intervention—it would at least necessitate frequent testing of patients at risk via biomarkers that are easily accessible (e.g. blood tests), very specific and sensitive and economically feasible.

Take the example of oncological diseases: Cancer is expected to be better treatable in a ‘pre-malignant’ stage, when first changes at the molecular level are detectable, but cancer cells have not yet developed all the traits that make them dangerous. However, not every precancerous lesion develops into the full clinical picture of a cancer. Furthermore, since the development of a disease is a continuum, it is not entirely clear at what point in time interventions can be classified as ‘early’. Does the concept already apply to the treatment of cancer precursors or only if the cells treated have clearly malignant features? Some authors apply ‘disease interception’ as a broad term for all approaches that reduce cancer risk—from primary prevention to early intervention (Beane *et al.*, 2017). Others prefer a more narrow definition like the one proposed above and use the term only for chemopreventive interventions as is envisioned for Alzheimer’s disease research (Lippman *et al.*, 2018). Whether and to what extent these terminological presuppositions are crucial for the ethical evaluation will be clarified below. A first conceptual caveat, however, can already be raised concerning the suitability of the term ‘interception’: if the term is used for interventions that ‘only’ reduce a risk, it is an unsuitable term because it promises that an already progressing disease is stopped. Purely risk-reducing measures belong to the category of primary prevention. If however the disease started but did not yet develop clinical symptoms, and all criteria included in the definition above are met, the intervention could be called ‘disease interception’ or, preferably, by a less suggestive name such as early and targeted secondary prevention by treatment. As will be shown, it is paramount that this narrow definition of the concept be carefully applied to avoid wrong expectations on the patient side.

## ‘Disease Interception’ in Light of the Four Principles of Biomedical Ethics

The following normative evaluation will be structured with recourse to the classical four principles of biomedical ethics introduced by Beauchamp and Childress: beneficence, nonmaleficence, autonomy and justice. These principles will serve as orientation for a general *ex ante* overview of some of the ethical challenges of early and targeted secondary prevention by treatment that can be anticipated.

## Beneficence: Benefit and Likelihood of Benefit

The benefit of early and targeted secondary prevention by treatment is to stop the progression of a dangerous preclinical disease. Three aspects have to be considered for the ethical utility evaluation of such an intervention: (i) the certainty with which the disease becomes symptomatic at all, (ii) the effectiveness of its prevention, and (iii) the threat or burden of the prevented disease. A classic example for a preventive intervention meeting all three criteria are patients with increased cardiovascular risk: high blood pressure and high blood lipids are treated by medication to prevent the sequela of cardiovascular stenosis like stroke or heart attacks. In one study, patients with coronary stenosis were able to reduce the risk of stroke by 33% and the risk of another cardiovascular event by 43% by regularly taking lipid-lowering drugs (statins) (Aboyans *et al.*, 2018).

This example shows that measures of chemoprevention are standard in medicine even if both the risk reduction and the likelihood of occurrence are far below 100%. Hence, it could be argued that the likely benefit of ‘disease interception’ would be even higher since it reacts not only to a high *risk* as the above cited primary prevention does but to *an already manifest* asymptomatic disease. But things are not as easy: The current medical and ethical discourse on statin use shows that refined biomarkers are warranted in order to reduce overtreatment especially of the elderly population (Mortensen *et al.*, 2019; Schade *et al.*, 2019). As has been said, the concept contains the strong claim that a clinically inapparent disease can be detected with a high degree of certainty. Such a strong claim needs to be grounded in the certainty that the preclinical disease really is on its way to develop its full clinical picture. The beneficence of the intervention is defined primarily by the danger that it averts. And this danger has to be precisely assessed. In this context, it may be necessary for future studies to delve deeper into the philosophy of medicine since the discussion, e.g. on cancer and on Alzheimer’s disease in their earliest stages has shown that the ontological and epistemological lines between risk and disease are quite blurred indeed (Schwartz, 2014; Reid, 2017; Schermer and Richard, 2018).

With regard to the principle of beneficence, timely and targeted prevention by treatment promises to be highly beneficial and morally allowed, if not imperative. This is especially true in comparison with already established measures of primary and secondary prevention since ‘disease interception’ focuses not on a more or less high risk but rather on the actual presence of the disease in its earliest stages, and promises an effective cure. However, this strong claim has to be sufficiently backed by future research.

## Nonmaleficence: Harm Potential and ‘Disease Interception’

It is obvious that every therapeutic intervention comes with certain side effects and that the benefits always need to be weighed against the risks. An example for this balancing is the selective estrogen receptor modulator tamoxifen that significantly reduces the incidence of breast cancer in women at high risk—42 women need to be treated in order to protect a single one against it (Cuzick *et al.*, 2013). At the same time, however, the risk of endometrial carcinoma doubles and, in addition, tamoxifen raises the risk of the occurrence of thromboembolic events. In order to assess the intervention’s harm potential, these significant side effects have to be taken into account.

More importantly, the potential harm of early and targeted secondary prevention by treatment can be further elucidated in the light of what has been termed ‘overdiagnosis’ and ‘overtreatment’ in public health ethics. Following Carter *et al.* (2016) overdiagnosis occurs when (i) a condition is identified and diagnosed in a population and (ii) this identification is scientifically correct, but (iii) the labeling and/or the intervention undertaken carries an unfavorable balance between benefits and harms. While this definition includes both the diagnosis and potential interventions triggered by the diagnosis, overtreatment focuses only on the treatment aspect. While there are many different definitions of the concept, most would agree that in overtreatment a disease that is not as harmful as expected is treated in such a way that the harms of the intervention outweigh the benefits (Klotz, 2012; DuMontier *et al.*, 2020). We know from the field of cancer screening that potential benefits need to be weighed against the harms of screening and need to be addressed explicitly in a shared-decision making with the patient. (Harris *et al.* 2014; Parker *et al.* 2017). It is therefore warranted that studies on early and targeted secondary prevention carefully record potential harms—which can pertain to physical, psychological harms and can encompass financial strain and opportunity costs.

A drug therapy for ‘disease interception’ will only be justified if both direct side effects and indirect risks with regards to other diseases are considered. With cancer and dementia being the main fields of research for ‘disease interception’, it could be argued that the benefits of stopping such serious diseases seem to substantially outweigh the potential harm. However, this will have to be informed by empirical evidence. The risk of overtreatment by ‘disease interception’ should be closely followed—especially in diseases that become symptomatic in old age so that patients might not live long enough to experience symptoms.

## Autonomy: Patient Preferences and Shared Decision-Making

The discussion about benefits and harms shows that the decision on ‘disease interception’ therapy requires a balanced decision-making process. Patients' preferences play a crucial role in this process. In view of interventions of ‘disease interception’ being tested in clinical research studies and entering routine care, we anticipate at least three decision-making scenarios in which these preferences are crucial: (i) patient preferences for ‘disease interception’ without other preventive alternatives; (ii) the preference for ‘disease interception’ measures if preventive alternatives, e.g. through a change of lifestyle exist; and (iii) the participation in clinical studies on new biomarkers and therapeutic approaches that are just on their way to generate the necessary evidence where the preference for or against risk information comes into play.

1. Patients‘ preferences and assessments play a decisive role in the weighing of short-term side effects against benefit and risk potentials distant in time. Interestingly, the uptake of tamoxifen therapy in our example has not been successful in the USA where it is approved. The preventive measure was accepted by less than 1% of eligible women (Waters *et al.*, 2010). The reasons have not been well-studied, but they range from inadequate risk communication and concerns about side effects to avoiding additional risks such as thrombosis and embolism. These plausible considerations make it clear that, as always when it comes to balancing decisions in medicine, a joint or participative decision-making according to a deliberative model is indispensable (Emanuel and Emanuel, 1992). This implies that physicians explain their expert knowledge in an understandable way while patients contribute their values and preferences with regard to the various options of therapy. For a successful process of shared decision-making, both parties must be well-informed: the doctor, who knows statistical data on prognosis and risk profiles and who needs to apply and communicate them to each individual case and the patient, who needs to understand and evaluate this information. It is safe to say that ‘disease interception’ approaches require a high level of education and communication on the medical side.
2. Patients will also need to choose between different approaches if ‘disease interception’ is offered in addition to existing preventive strategies, for example based on behavioral changes. Interestingly enough, the literature on the effects of behavioral change, e.g. in secondary cancer prevention, has also begun reframing it as ‘interception’ (Iyengar and Jones, 2019). The following conflict could ensue: Patients with impaired glucose tolerance for example, can actively act against the development of the full-scale picture of diabetes type 2 by weight loss—but taking an antidiabetic pill is often easier than changing one‘s lifestyle. Similarly, smokers are likely to continue smoking if the development of a preventive substance will succeed that can reverse and cure tobacco-smoke-related bronchial dysplasia at an early stage. Hence, the ‘disease interception’ pill might be the easier way than smoking cessation (Lippman *et al.*, 2018). We know today that an estimated 35% of the expected cases of cancer worldwide are caused by avoidable risk factors—especially smoking, followed by overweight, lack of exercise and heavy drinking (Danaei *et al.*, 2005). This should, however, not lead to what has accurately been termed a ‘moral responsibilisation’ of health: individuals should not sweepingly be blamed for being fully or partly responsible for the lifestyle-induced disease they are suffering from (Brown *et al.*, 2019). It is clear that the above-mentioned risk factors are dependent of the socio-economic context and not just the result of self-imposed lifestyles (Levy, 2019). In light of the responsibility discussion in public health ethics, ‘disease interception’ by medication might also present a chance for socio-economically disadvantaged patients: if behavioral changes are not feasible for them, they still have an alternative at hand. Thus, ‘disease interception’ could enable what Vansteenkiste *et al.* (2014) have envisioned as a ‘fresh start’ for patients with lifestyle-induced diseases. It could be argued that ‘disease interception’ poses both a threat and an opportunity when weighed against the alternative of behavioral changes.
3. Thirdly, and perhaps most relevant at the moment, the introduction of ‘disease interception’ interventions rests upon extensive research on new biomarkers and therapeutic approaches. This means that a better understanding of the risks and the discovery of relevant biomarkers precede the development of concrete treatment options. It is precisely this phase of research—where disease prediction becomes better and still no treatment option is available—that requires us to respect the preferences of the potentially affected to learn or not to learn about their risk for developing a disease.

We know from human genetics that the value of knowing one’s risk for disease is considered to be very ambivalent, especially if the course of disease cannot be influenced by either early detection or treatment. For example, in the case of Huntington’s Chorea only about one in five affected family members would like to know if they are carriers of the gene in order to better plan their lives. The other four out of five want to preserve the feeling of an open future and shy away from an experience of informed powerlessness (Taylor, 2004; Robins Wahlin, 2007). Accordingly, human genetic counseling is one of the areas of medicine in which a ‘right not to know’ has been established. It is a defense against all information that could irritate the autonomous person and the development of their personality. Here, autonomy is understood as an activity of self-creation, in which the person concerned may also repel information that restricts human self-design (Andorno, 2004). This shows once again the high demands placed on risk communication and the information requirements of potential study participants who are advancing disease-interception research.

In order to foster patient autonomy, patient preferences should be discussed and finally incorporated into a shared decision-making process. Especially in the case of ‘disease interception’ interventions that are full of promises, the potential benefit and harm as well as preventive alternatives should be clearly communicated. This should also include a reflection on the language used to inform the patient and its potential hidden biases. As argued above, the innovative and attractive term ‘disease interception’ that is essentially a metaphor and not a medical term already includes a strong positive bias. The principle of patient autonomy underlines how crucial it is that such biases be avoided in order to achieve informed consent and a balanced doctor–patient relationship.

## Justice: Fair Access to and Use of Drug Risk Reduction and Prevention

When discussing ‘disease interception’ as an alternative to behavioral changes, it is clear that this is a question not only of autonomy but also of justice: Is the choice between behavioral modification and the ‘disease interception’ drug left to the individual or should measures of ‘conventional’ prevention and ‘disease interception’ be coordinated with one another in a compulsory manner? How can justice be ensured in this process?

In a collectively funded health care system, individuals could be required to contribute to their own risk reduction before resorting to ‘disease interception’, which we can expect to be expensive when it enters the health care market. It would be conceivable that e.g. at least one program for risk avoidance must have been credibly attempted and performed before chemoprevention can be prescribed. On the other hand, vulnerable populations socioeconomically disadvantaged by higher exposure to risk constellations and lower health literacy should not be disadvantaged and denied access to new ways of ‘disease interception’. Providing vulnerable populations with it could even be a way of alleviating the disadvantages they suffer.

From the perspective of the principle of justice, there are a number of concerns which have to be addressed when establishing ‘disease interception’ in the health care system. It is not so much ‘disease interception’ itself, but as it were, its ‘competition’ with other preventive services that raises these questions. When providing patients with ‘disease interception’, a healthy balance has to be found between requiring them to actively engage in behavioral changes as a means of primary prevention and respecting the limits of vulnerable patients from socioeconomically disadvantaged groups to do so.

## The ‘Healthy Ills’? Social Implications of a Preponed Concept of Disease

Broadening the scope of this *ex ante* assessment further, a social aspect of ‘disease interception’ can be identified, that is relevant to both the individual and our health care system in general: the changes that knowledge about having a clinically inapparent disease brings about for the concepts of disease and illness. Again, from the counseling practice in human genetics it is well known that the information that one carries a significantly increased risk for a disease is often experienced as a shock and a major biographical incision. There is therefore a danger that genetic risk knowledge will suddenly ‘make’ healthy people ill—a danger that has been pointed out already in the 1990s and that is now all the more realistic (Hubbard, 1993). ‘Disease interception’ poses a similar risk that is even more imminent because it addresses not a risk but the present, though clinically inapparent, disease thereby creating ‘healthy ills’: These patients who feel as if they already have the full picture of the disease could then expect the same amount of attention and consideration for their illness that our health care system has so far only shown those who are ‘actually diseased’ i.e. that already show symptoms. With the general introduction of ‘disease interception’ the oxymoron of the ‘healthy ill’ becomes a social reality. Again, these are questions that are well known from the context of genetics and from the increasing discourse on overdiagnosis and overtreatment mentioned above. Among others, Hofmann (2019) has already discussed the problem of overdiagnosis and patients with a disease that do not yet experience any symptoms. These aspects become a special urgency and should be closely monitored in the context of early and targeted secondary prevention by treatment.

The concept of ‘disease interception’, if taken literally, goes beyond the probability calculus and seeks to find indicators that already prove early stages of a disease in order to intervene before the disease becomes symptomatic. Especially for Alzheimer’s dementia, this seems necessary and first biomarkers that indicate early degradation processes are already being identified. Here, the arguments that speak in favor of ‘disease interception’ are very convincing. Just as we treat patients with increased cardiovascular risk or risk of cancer recurrence to reduce the risk, we would want to treat people with Alzheimer’s disease before the disease is fully manifest—provided that the above-mentioned criteria are adequately met and taken into account.

## Conclusion

The aim of nipping diseases in the bud even before the patient displays symptoms is very promising and at first glance almost revolutionary. Given the name ‘disease interception’ it becomes even more attractive and the emerging debate shows that various medical fields could be profoundly influenced by the ‘new’ concept.

Faced with such scientific enthusiasm it is necessary to take a step back and evaluate the ethical and social implications of ‘disease interception’. Already this early in the debate, it can be concluded that the term ‘disease interception’ is far from a precise scientific decision. It remains a commercially motivated metaphor for a measure that is essentially a specific form of secondary prevention. The term ‘disease interception’ refers to a form of secondary prevention by medication applied in a short interception window and intended to prevent a disease that is developing but still clinically inapparent from developing its full clinical picture. A more accurate term that contains the elements of this definition could therefore be early and targeted secondary prevention by treatment. This clarification is not just terminological hair-splitting but already has ethical implications: In order to ensure a shared decision-making process that respects patient autonomy, it is crucial that potentially biased metaphors such as ‘disease interception’ make way for a more neutral scientific terminology as well as a clear and narrow definition of the concept. For the sake of patient information the definition of ‘disease interception’ as early and targeted secondary prevention by treatment should be disclosed and sufficiently explained.

Furthermore, future research into this new form of prevention has to take into account not only the difficult medical question how to detect a clinically inapparent disease but also the fundamental ethical and social question raised in the light of the four principles of biomedical ethics by Beauchamp and Childress, *if* and *when* such an intervention is justifiable and what consequences it has for the individual patient and society as a whole.

## Conflict of Interest

E.C.W. was invited as ethics expert in a workshop and open house seminar on ‘disease interception’ organized by Janssen Cilag and received personal fees for her contribution outside of the submitted work. She also contributed a chapter on ethical aspects of disease interception in a book series on health service research issues that was partly sponsored by Janssen Cilag. J.N. was invited to the above-mentioned event as a participant.

## Footnotes

1 So far, the concept of ‘disease interception’ has almost exclusively been applied to treatment by medication. We will therefore often speak of ‘disease interception’ as a form of chemoprevention while at the same time acknowledging the possibility that e.g. certain surgical interventions could fulfill the criteria of ‘disease interception’ introduced above. The term ‘treatment’ is broad enough to cover both of these possibilities and at the same time specific enough to distinguish ‘disease interception’ from other measures of secondary prevention primarily focusing on screening and identifying diseases.
